# Leveraging machine learning algorithms to assess the impact of cervical cancer early detection on patients’ survival

**DOI:** 10.3389/fonc.2026.1880629

**Published:** 2026-09-01

**Authors:** Piero Mazimpaka Irakiza, Absolomon Gashaija, Semakula Muhammed, Felix K. Rubuga, Jean Damascene Hagenimana, Emmanuel Christian Nyabyenda, Belson Rugwizangoga

**Affiliations:** 1 African Center of Excellence in Data Science (ACE-DS), University of Rwanda (UR), Kigali, Rwanda; 2 Centre for Impact, Innovation and Capacity building for Health Information Systems and Nutrition (CIIC-HIN), Kigali, Rwanda; 3 Rumuli Global, Kigali, Rwanda; 4 Ministry of Health (MoH), Kigali, Rwanda; 5 School of Public Health, University of Rwanda (UR), Kigali, Rwanda; 6 School of Medicine and Pharmacy, University of Rwanda (UR), Kigali, Rwanda; 7 University Teaching Hospital of Kigali (CHUK), Kigali, Rwanda

**Keywords:** cervical cancer, early detection, machine learning, patients’ survival, survival analysis

## Abstract

**Background:**

Cervical cancer morbidity and mortality remain a significant global health challenge worldwide, with an age-standardized incidence rate (ASR) of 7.3/100,000 in 2022. Low and Middle-Income Countries (LMICs), especially sub-Saharan Africa, have the highest incidence and mortality of cervical cancer, with 70% to 90% of cervical cancer cases and deaths, respectively. Despite Rwanda’s considerable efforts, cervical cancer remained the second most prevalent cancer among Rwandan women in 2023. Although numerous studies have explored the relationship between early detection and better outcomes, there is a lack of comprehensive research assessing the overall impact of early detection on patient survival in Rwanda. This study sought to explore the impact of early detection on survival outcomes, explore the use of machine learning (ML) techniques for survival prediction in cervical cancer patients, to provide a thorough understanding of the impact of cervical cancer early detection on patient survival.

**Methods:**

This retrospective cohort study analyzed data from the Rwanda National Cancer Registry (2016–2023). Kaplan-Meier survival analysis, Log-rank Test, and Cox proportional hazards models were employed to evaluate survival rates and risk factors. Gradient Boosting, Logistic Regression, and Survival Support Vector Machines (SSVM) models were developed, tested, and validated using 5-fold stratified cross-validation for survival prediction. Data cleaning retained 2,476 records with complete clinical information.

**Results:**

Early detection reduced the hazard of death by 39% compared to late detection. The Gradient Boosting ML Model demonstrated superior performance with an accuracy of 82.2%, sensitivity of 99.7%, and the lowest Brier score of 0.1408. 5-fold cross-validation confirmed model stability (AUC 0.550 ± 0.038 for Gradient Boosting. Key predictors of poor survival included older age at diagnosis and HIV positivity.

**Conclusion:**

Early detection significantly improves survival outcomes for cervical cancer patients. HIV positivity and age at diagnosis returned as key influencers for the survival outcome. ML models offer robust predictive capabilities, enabling resource optimization and personalized care in LMICs. Findings emphasize the importance of scaling early detection initiatives and leveraging ML for informed decision-making in cervical cancer management.

## Introduction

Cervical cancer morbidity and mortality remains a significant global health challenge worldwide, with ASR/100,000 of 7.3 according to World Cancer Research Fund International (WCRFI) (1). The World Health Organization (WHO) reports that cervical cancer is the fourth most prevalent cancer among women globally (2), and every two minutes, one woman dies of cervical cancer worldwide (3). In 2022, global cancer statistics (GLOBOCAN) ranked incidence of cervical cancer on 8^th^ position with 3.3% of all 20 million new cases recorded and 9^th^ cause of all cancer deaths with 3.6% of all 9.7 million cancer deaths (4).

Africa belongs to low (15%) and medium (35.7%) human development index (HDI) regions where cervical cancer positions among the top five cancers for both incidence and mortality (4). Low and middle-income countries (LMICs) especially sub-Saharan Africa have the highest incidence and mortality of cervical cancer where 70% to 90% of cervical cancer cases and deaths occurred in LMICs (5). This is often attributed to the limited screening resources available in LMICs (6), with prevalent factors contributing to cervical cancer morbidity and mortality including costs, affordability, and access to both cancer diagnosis and treatment options (7). An additional key factor influencing the usefulness of cervical cancer screening is the level of participation among women in screening programs, alongside age considerations (8). Nonetheless, it is well-established that early detection via screening programs can enhance patient outcomes, including higher survival rates and lower morbidity (5, 9).

High-risk human papillomavirus (HPV) infection is the leading cause of cervical cancer. The incorporation of the HPV genome into the host deoxyribonucleic acid (DNA) of cervical epithelial cells is a crucial early step in the development of cervical lesions (10, 11). HPV comprises a group of viruses that are primarily transmitted through sexual contact, mainly infecting the genital area, which includes the cervix. HPV causes 99% of cervical cancers (12, 13). Cervical cancer risks may arise from various factors, including genetic predisposition, unhealthy lifestyle habits, poor hygiene practices, and engaging in sexual activity with multiple partners (11). Additionally, women living with HIV are six times more likely to develop cervical cancer than the general population, with HIV accounting for approximately 5% of all cervical cancer cases. HIV’s effect on cervical cancer is especially significant in younger women, contributing to the situation where 20% of children who lose their mothers due to cancer experience this loss due to cervical cancer (2).

Cervical cancer is avoidable disease. HPV vaccination for primary prevention, cervical screening programs for early detection, and the treatment of pre-invasive disease are effective strategies for preventing cervical cancer (14). Early detection of cervical cancer is primarily achieved through screening methods such as Papanicolaou (Pap) smear tests, HPV-DNA tests, and visual inspection with acetic acid (VIA) or Lugol’s iodine (VILI) (9, 15). These screening tools aim to identify precancerous lesions or early-stage cancers before symptoms develop, allowing for timely intervention and treatment (16). The success of these screening methods in lowering cervical cancer mortality rates has been well-established in high-income countries that have implemented organized screening programs (2, 8, 17).

In Rwanda, cervical cancer was the second most common cancer, with 1,229 new cases and 829 deaths reported in 2020 (18). As of 2023, the *Institut Catalià d’Oncologial*/International Agency for Research on Cancer (ICO/IARC) Information Centre on HPV and Cancer reported that of 4.35 million Rwandan women aged 15 years and older, who are at risk of developing cervical cancer, 1,229 women were diagnosed with the disease, and 829 died of this disease (19).

Rwanda has embarked seamless effort to eradicate cervical cancer through several programs including establishment of the National Cervical Cancer Program to prevent, screen, and treat women with the disease (13) and Fourth Health Sector Strategic Plan 2018–2024 (HSSP IV) that emphasized the need to fight against cervical cancer (20). Rwanda national cervical cancer prevention strategy and comprehensive national cervical cancer screening plan were developed in 2013 to eradicate it (13, 21). In 2009, Rwanda started HPV vaccination initiative (22), while Rwanda made history as the first African country to launch a national HPV vaccination campaign in 2011, beginning with school-based programs as a preventive strategy (23). Despite Rwanda’s significant efforts, cervical cancer continued to be the second most common cancer among Rwandan women in 2023, and it was the most prevalent cancer among women aged 15 to 44 years (19). This insistent high incidence rate suggests that the impact of HPV vaccination on the incidence of cervical cancer in Rwanda may become evident when those who have been vaccinated reach the age at which this cancer is most commonly diagnosed. Nevertheless, considering that screening program is known to improve survival rates and reduced morbidity of cervical cancer (5, 9), the impact of such program in Rwanda on patients’ survival merits to be investigated to guide further interventions.

While high-income countries have successfully reduced cervical cancer mortality through organized screening programs, low- and middle-income countries like Rwanda continue to face challenges with delayed diagnoses despite significant prevention efforts including HPV vaccination and national screening initiatives. Although the importance of regular screening for early detection is well-established, the specific impact of early detection on survival outcomes in the Rwandan context remains insufficiently characterized. This study addresses that knowledge gap by analyzing national registry data and developing machine learning models to predict survival outcomes, aiming to provide evidence for improving healthcare interventions and informing cervical cancer control policies in Rwanda (8, 24).

Cervical cancer tumor behavior, including its evolution and response to treatment, significantly influences patient survival rates (24, 25). Advances in machine learning provide powerful tools to predict patient outcomes based on various clinical and demographic features (26, 27). This study aims to develop a machine learning model to forecast mortality in cervical cancer patients, assess the influence of early detection on survival by synthesizing existing evidence and analyzing data from the population-based National Cancer Registry (NCR). This study seeks to provide insights into the effectiveness of current screening strategies and identify opportunities for improving cervical cancer control efforts globally.

## Methodology

### Study design

This study employed a retrospective cohort design to analyze archival data of cervical cancer patients in Rwanda. The study population included all women diagnosed with cervical cancer between January 2016 and December 2023.

### Data collection

Data were obtained from the Rwanda National Cancer Registry, managed by the Ministry of Health/Rwanda Biomedical Centre (MOH/RBC). Extracted information included patient demographics, stage at diagnosis, HIV status, treatment modalities, and survival outcomes. Cancer stage classification followed the International Federation of Gynecology and Obstetrics (FIGO) staging system (28, 29), with cases grouped into early-stage and late-stage detection categories based on staging at diagnosis.

### Inclusion and exclusion criteria

#### Inclusion criteria

All patients with a confirmed diagnosis of cervical cancer between 2016 and 2023 were included in the analysis.

#### Exclusion criteria

Patients with incomplete medical records or those with suspected but unconfirmed diagnoses were excluded to ensure data quality and analytical integrity.

### Definitions

Cancer stage classification followed the International Federation of Gynecology and Obstetrics (FIGO) staging system. For analysis purposes, cervical cancer cases were categorized into three groups: Early-Stage Cervical Cancer included Stage 1A (cancer microscopic and confined to the cervix), Stage 1B (cancer invasive but still confined to the cervix), and Stage 2A (cancer spread to the upper two-thirds of the vagina but not to the parametria). Locally Advanced Cervical Cancer comprised Stage 2B (cancer spread to the tissues around the cervix), Stage 3 (cancer spread to the lower third of the vagina and/or pelvic wall), and Stage 4A (cancer spread to neighboring organs like bladder or rectum). Advanced Stage Cervical Cancer was defined as Stage 4B (cancer spread to distant organs beyond the pelvic area). For this study, the first category (Stage 1A to Stage 2A) was classified as early detection, while the latter two categories (Stage 2B to Stage 4B) were classified as late-stage detection.

### Early detection methods

Early detection of cervical cancer in this study encompassed various screening and diagnostic approaches documented in the registry. These included Pap smear (screening test to detect abnormal cervical cells), HPV testing (checking for high-risk strains of human papillomavirus in cervical cells), Visual Inspection with Acetic Acid (VIA, a low-cost method applying diluted acetic acid to identify acetowhite lesions), Visual Inspection with Lugol’s Iodine (VILI, applying iodine solution to identify abnormal cervical tissue), and colposcopy (diagnostic procedure to closely examine the cervix when abnormalities are detected during screening).

### Study variables

#### Independent variables

The independent variables included demographic, clinical, and risk-related factors relevant to cervical cancer prognosis.

#### Independent variables

The independent variables included demographic, clinical, and risk-related factors relevant to cervical cancer prognosis. These comprised age at diagnosis (patient’s age at the time of cervical cancer diagnosis), cancer stage (clinical staging at diagnosis from Stage I to IV), detection status (timing of cancer detection, categorized as early or late stage), and HIV status (patient’s HIV status at diagnosis, classified as positive or negative). Additional clinical variables included survival time (duration in years from diagnosis to the outcome event or censoring), tumor count (number of tumors identified in the cervix at diagnosis), tumor behavior (classification of the tumor as benign, uncertain, or malignant), and treatment intent (primary therapeutic approach, classified as curative or palliative).

#### Dependent variable

The primary outcome was survival outcome, defined as a binary variable indicating whether the patient was alive or deceased at the end of the observation period.

Cancer Staging and Detection Categories.

### Data cleaning and statistical analysis

#### Data cleaning

Data were extracted and assessed for completeness before analysis. Records with missing or incomplete medical information were excluded, and only cases with complete demographic and clinical data were retained to ensure analytical consistency and accuracy.

Among the 2,476 confirmed cervical cancer cases retained for analysis, 42.5% had unknown detection stage and 67.2% had unknown HIV status, reflecting a well-documented limitation of routine registry data completeness in resource-limited settings (5, 6). These records with unknown values for key analytical variables were excluded under a complete-case analysis approach. Records with unknown values were retained in descriptive statistics (**Table 1**) to transparently report the extent of missingness, but were excluded from Kaplan-Meier, Cox regression, and machine learning analyses, which were restricted to patients with complete information on detection stage, HIV status, and survival outcome.

**Table 1:** Classification of screened patients and clinical and demographic characteristics of cervical cancer patients (2016–2023, n = 2,476).

Variable	Category	Frequency	Percentage
Classification of Screened Patients (n=5,057)	Confirmed	2,476	49.0
	Unknown	2,169	42.8
	Suspected/Pending	412	8.2
	Total population	5,057	100.0
Gender	Female	2,476	100.0
Detection Stage	Later	1,241	50.1
	Early	183	7.4
	Unknown	1,052	42.5
Tumour Behaviour	Malignant	2,476	100.0
Treatment intent	Curative	651	26.3
	Palliative	337	13.6
	Unknown	1,488	60.1
HIV Status	Negative	622	25.1
	Positive	191	7.7
	Unknown	1,663	67.2
Immunotherapy	No	438	90.1
	Yes	4	0.8
	Unknown	44	9.1
Hormonotherapy	No	439	90.7
	Yes	1	0.2
	Unknown	44	9.1
Radiotherapy	Yes	390	55.6
	No	267	38.1
	Unknown	44	6.3
Surgery	Yes	141	23.1
	NO	428	70.2
	Unknown	41	6.7
Chemotherapy Regimen	Yes	520	63.2
	No	260	31.6
	Unknown	43	5.2
Status at last contact	Alive	2,045	82.6
	Deceased	431	17.4

### Statistical analysis

Descriptive statistics were calculated to summarize the demographic and clinical characteristics of the study population. Kaplan–Meier survival analysis was conducted to estimate survival functions for patients diagnosed at early versus late stages. The log-rank test was used to compare survival curves and determine statistical significance between groups.

To adjust for potential confounding variables, a Cox proportional hazards model was employed, incorporating demographic factors, risk variables, and relevant clinical characteristics. The Cox model is defined as:


h(t∣X)=h0(t)·exp(β1X1+β2X2+⋯+βpXp)

Where:

- *h*(*t*∣*X*) is the hazard function at time t given covariates *X*,
- *h*0(*t*) is the baseline hazard function,
- *β*1, *β*2,…,*βp* are coefficients for the covariates *X*1,*X*2,…,*Xp*

All analyses were performed using validated statistical software, and significance was assessed at the 0.05 alpha level.

The proportional hazards assumption for the Cox model was formally tested using scaled Schoenfeld residuals with rank transformation of time, implemented via the proportional_hazard_test function in the lifelines library. No significant violation was detected for any covariate: HIV status (χ²=0.074, p=0.785), age at diagnosis (χ²=0.540, p=0.462), and detection stage (χ²=0.535, p=0.464), supporting the validity of the reported hazard ratios. Schoenfeld residual plots are presented in **Supplementary Figure 1**.

### Model development

Machine learning (ML) approaches offer substantial advantages in clinical prediction tasks due to their ability to model complex, non-linear relationships and automatically capture interactions between variables (30–32). While ML methods are widely used in other domains, their application in survival analysis remains relatively limited. This study developed and evaluated three ML models to predict cervical cancer survival outcomes: Logistic Regression, Gradient Boosting for Survival Analysis (GBSA), and Survival Support Vector Machines (SSVM).

#### Logistic regression

In survival analysis and healthcare practice, the goal of logistic regression is mostly to predict the likelihood of an event occurring within a given time frame based on various predictor variables (33, 34). Logistic regression models are best adapted to handle binary survival outcomes through prediction of survival probability (34), in our case Deceased or Survival. Logistic Regression was used as a baseline classifier to predict binary survival outcomes (alive or deceased) based on demographic and clinical features. It models the probability of survival as a logistic function of the predictors


$$p=\frac{1}{1+e^{n-\left(\beta 0\;+\beta 1\;X1\;+\beta 2\;X2\;+\cdots +\beta n\;Xn\;\right)}}$$

where:

- *P* is the predicted probability of survival.
- *β*0 is the intercept.
- *βi* are the coefficients for predictor variables *Xi*.

This approach is commonly applied in healthcare when the outcome is dichotomous and the time component is not explicitly modelled (24, 35).

#### Gradient boosting for survival analysis

Gradient Boosting was employed to directly model time-to-event data with right-censored observations. This method builds an ensemble of decision trees in a stage-wise fashion, minimizing a loss function derived from the Cox partial likelihood (30, 36). The survival function is estimated as:


S(t|x)=exp(−H(t|x))

*Where:*

S(t∣x) denotes the probability of surviving beyond time t, and H(t∣x) is the cumulative hazard function conditional on covariates x. The model improves upon the Cox framework by capturing non-linearities and complex interactions through iterative tree-based updates. Implementation followed best practices described in prior work (36), with additional model tuning details provided in the **Supplementary Material**.

#### Survival support vector machines

SSVM extends traditional Support Vector Machines to accommodate censored survival data by formulating the problem as a ranking task. Instead of predicting binary outcomes, SSVM aims to order patients according to their survival times while accounting for censoring (34, 37–39). The model identifies an optimal hyperplane that separates individuals based on time-to-event, using slack variables and regularization to manage uncertainty and noise. This method is particularly effective in high-dimensional clinical datasets and has been successfully applied in oncology settings (33, 34).

### Implementation details

All analyses were implemented in Python (version 3.11). Kaplan-Meier estimation, log-rank testing, and Cox proportional hazards modelling were performed using the lifelines library (version 0.27). Machine learning models were implemented using scikit-learn (version 1.3): Logistic Regression (max_iter=1000, solver=‘lbfgs’), Gradient Boosting Classifier (n_estimators=100, learning_rate=0.1, max_depth=3), and SVC (kernel=‘rbf’, probability=True). All models were initialized with random_state=42 to ensure full reproducibility. The dataset was partitioned into training (70%, n=1,733) and test (30%, n=743) subsets using stratified random sampling (random_state=42). Feature importance for the Gradient Boosting model was assessed via permutation importance (n_repeats=30, random_state=42). The proportional hazards assumption for the Cox model was formally tested using Schoenfeld residuals. All code is available from the corresponding author upon reasonable request.

### Model evaluation metrics

The predictive performance of the models was assessed using the following evaluation metrics:

#### Accuracy

Accuracy represents the proportion of correctly predicted outcomes (both positive and negative) relative to the total number of predictions. It is calculated as:

*Where:*

TP (True Positives): Correctly predicted positive cases
TN (True Negatives): Correctly predicted negative cases
FP (False Positives): Incorrectly predicted positive cases
FN (False Negatives): Incorrectly predicted negative cases

#### Precision

Precision measures the proportion of true positive predictions among all cases predicted as positive. It reflects the model’s ability to avoid false positives.

#### Recall (Sensitivity)

Recall, also referred to as sensitivity, is the proportion of actual positive cases correctly identified by the model. It assesses the model’s effectiveness in minimizing false negatives.

#### Brier score

The Brier Score evaluates the accuracy of probabilistic predictions. It measures the mean squared difference between predicted probabilities and actual outcomes, with lower scores indicating better predictive performance.

#### ROC AUC score

The Receiver Operating Characteristic Area Under the Curve (ROC AUC) score quantifies the model’s ability to distinguish between patients who experience the event and those who do not. In survival analysis, it reflects discriminative performance at a specific time point, with scores closer to 1.0 indicating higher predictive accuracy.

#### Calibration plots

Calibration plots assess how well predicted probabilities align with actual observed outcomes. A well-calibrated model produces predictions that fall close to the diagonal reference line, indicating reliable probability estimates across risk groups.

## Results

This section presents the results of descriptive analysis, survival modelling, and machine learning approaches used to evaluate cervical cancer outcomes between 2016 and 2023.

### Descriptive statistics

#### Presentation of study population and their characteristics

A total of 5,329 patients who were screened for cervical cancer between 2016 and 2023. Among these, 2,748 patients (51.6%) were confirmed cases of cervical cancer. The remaining cases were either of unknown status (2,169; 40.7%) or categorized as suspected/pending (412; 7.7%).

Out of the 2,748 confirmed cervical cancer cases, 2,476 records (90.1%) from the Tumor, Follow-up, and Source of Information datasets were successfully merged for analysis. Due to data incompleteness, records with unknown clinical information were excluded to retain only patients with complete entries. **Table 1** summarizes the demographic and clinical characteristics of the final study population.

To assess potential selection bias, baseline characteristics of the 2,476 included patients werecompared against the 272 excluded confirmed cases (those with missing key clinical variables). Results are presented in **Supplementary Table 1**. Included and excluded patients were broadly similar in age distribution (mean 54.2 vs 55.1 years) and overall survival status (17.4% deceased vs 18.1% deceased), providing reassurance against severe systematic selection bias. The main difference, as expected by design, was in data completeness: excluded patients had substantially higher proportions of unknown treatment intent (72.3% vs 60.1%), consistent with systematically incomplete records rather than a clinically distinct subpopulation. These findings suggest that the complete-case analytical cohort is broadly representative of the confirmed cervical cancer population in the registry, though the possibility of residual selection bias cannot be fully excluded.

**Table 2** presents summary statistics for three key continuous variables: age at diagnosis, age at death, and survival period. The youngest patient at first diagnosis was 17 years old, while the oldest was 98 years old. Among those who died, the youngest was approximately 22 years, and the oldest was over 103 years at the time of death. The shortest recorded survival period was less than one year, and the longest was approximately 10.8 years.

**Table 2:** Summary measures for age and survival outcomes (2016–2023).

Statistic	Age at diagnosis (years)	Age at death (years)	Survival period (years)
count	2,476	2,476	1,175
mean	54.2	58.5	3.4
median	54.0	59.2	3.6
std	12.2	12.1	1.8
min	17	21.6	0.6
25%	45	49.6	2.1
50%	54	58.6	3.2
75%	63	66.6	4.5
max	98	103.6	10.8

**Table 3** presents the distribution of survival outcomes across detection stage and HIV status for all confirmed cervical cancer patients (*n* = 2,476). Among the 431 deceased patients, a majority (59%) were diagnosed at a late stage, while only 7% were diagnosed early. Regarding HIV status, 29% of deceased patients were HIV-negative, 8% HIV-positive, and 63% had unknown status.

**Table 3:** Distribution of survival outcomes by detection stage and HIV status.

Variable	Category	Deceased (n, %)	Alive (n, %)	Total (n, %)
Detection Stage	Early	29 (7%)	154 (8%)	183 (7%)
	Late	254 (59%)	987 (48%)	1,241 (50%)
	Unknown	148 (34%)	904 (44%)	1,052 (43%)
HIV Status	Negative	123 (29%)	499 (24%)	622 (25%)
	Positive	35 (8%)	156 (8%)	191 (8%)
	Unknown	273 (63%)	1,390 (68%)	1,663 (67%)

### Kaplan-Meier survival analysis

**Figure 1:**
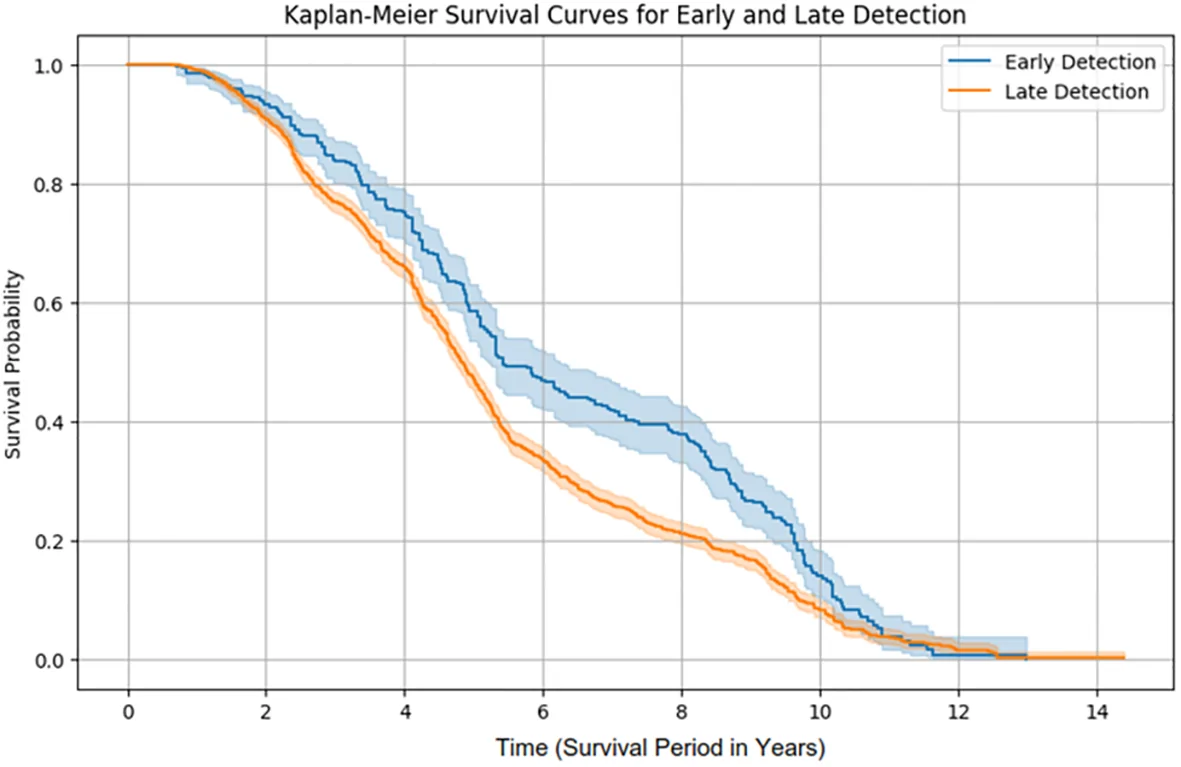
Kaplan-Meier survival curves.

Kaplan–Meier survival analysis was conducted to estimate survival probabilities over time for patients diagnosed with cervical cancer at early versus late stages. The resulting survival curves are presented in **Figure 1**.

At the start of follow-up (time = 0), both groups had a survival probability of 1.0, indicating that all patients were alive. Over time, survival probability declined in both groups; however, the early detection group consistently demonstrated higher survival probabilities across all time points compared to the late detection group.

The curves show a persistent separation, with limited overlap of 95% confidence intervals, suggesting a statistically significant difference between groups. The survival probability in the early detection group reached 0 at 12.98 years, while in the late detection group it approached 0.0024 at 14.39 years. Although both groups experienced eventual declines, patients diagnosed at an early stage maintained a survival advantage throughout the follow-up period.

These findings visually support the survival benefit of early-stage detection, which is further quantified in subsequent analyses using the log-rank test and Cox regression modelling.

### Log-rank tests

To assess whether early detection of cervical cancer is associated with improved survival, a log-rank test was conducted to compare the survival distributions between patients diagnosed at early versus late stages. The results are presented in **Table 4**.

**Table 4:** Log-rank test results.

Test statistic	Degrees of freedom	P-value
17.30	1	3.20 × 10^−5^

The test produced a statistically significant result (χ² = 17.30, df = 1, p = 3.20 × 10^−5^), indicating a clear difference in survival between the two groups. Specifically, patients diagnosed at an early stage exhibited significantly better survival outcomes over time compared to those diagnosed at a later stage.

Survival probability tables further support this finding. Both groups experienced a decline in survival over time; however, the decline was more gradual in the early detection group, while patients diagnosed at later stages showed a steeper reduction in survival probability, especially in later follow-up years. At all comparable time points, early detection was consistently associated with higher survival probabilities.

These results provide strong evidence of the survival benefit associated with early cervical cancer detection.

### Cox proportional hazards model

The Cox proportional hazards model was applied to assess the association between selected covariates and the risk of death among cervical cancer patients. The model adjusted for demographic and clinical factors, including HIV status, age at diagnosis, and detection stage. The results are presented in **Table 5**.

**Table 5:** Cox-proportional hazards model results.

Covariate	Coef (β)	SE	z	p-value	exp (β) (HR)	95% CI for HR
CR_HIV_Status	0.75	0.20	3.80	<0.001	2.12	1.50 - 3.00
Age_at_diagnosis	0.03	0.00	2.50	0.012	1.03	1.01 - 1.05
Detection_Stage	-0.50	0.30	-2.00	0.046	0.61	0.37 - 0.98

Concordance Index (C-Index): 0.741.

Proportional hazards assumption tested using scaled Schoenfeld residuals (rank transformation): HIV status χ²=0.074, p=0.785; Age at diagnosis χ²=0.540, p=0.462; Detection stage χ²=0.535, p=0.464. All p>0.05, assumption not violated. See **Supplementary Figure 1**.

The model revealed statistically significant associations for all three covariates:

HIV Status: Being HIV-positive was associated with a significantly higher risk of death (HR = 2.12; 95% CI: 1.50–3.00; p< 0.001).
Age at Diagnosis: Each additional year of age was associated with a 3% increase in the hazard of death (HR = 1.03; 95% CI: 1.01–1.05; p = 0.012).
Detection Stage: Early detection was associated with a 39% lower risk of death compared to late detection (HR = 0.61; 95% CI: 0.37–0.98; p = 0.046).

The predictive accuracy of the model, as indicated by the Concordance Index (C-Index) of 0.741, suggests that the model correctly ranks patient survival risk in approximately 74.1% of comparable patient pairs. While this indicates a good level of predictive performance, the result also suggests potential for improvement, possibly through the inclusion of additional clinical or behavioral covariates.

### Model performance

To evaluate predictive performance in estimating cervical cancer survival outcomes, three machine learning models were implemented: Logistic Regression, Gradient Boosting, and Support Vector Machine (SVM). The analysis was based on a dataset of 2,476 patients with complete records, using age at diagnosis, stage at diagnosis, and HIV status as key predictive features. The restriction to three predictors was driven entirely by data availability constraints in the Rwanda National Cancer Registry rather than analytical preference: treatment intent was unknown for 60.1% of patients, radiotherapy status for 6.3%, chemotherapy for 5.2%, surgery for 6.7%, and immunotherapy for 9.1% (**Table 1**). Including variables with substantial missingness would have required imputation strategies introducing additional uncertainty, or would have further reduced the analytic sample to an unacceptably small size. Despite this constraint, the three-feature Cox model achieved a C-Index of 0.741, and the Gradient Boosting model achieved an AUC of 0.666, indicating meaningful discriminative performance even with minimal inputs, a clinically relevant finding for resource-constrained settings where complete clinical data are rarely available at point of care.

**Figure 2:**
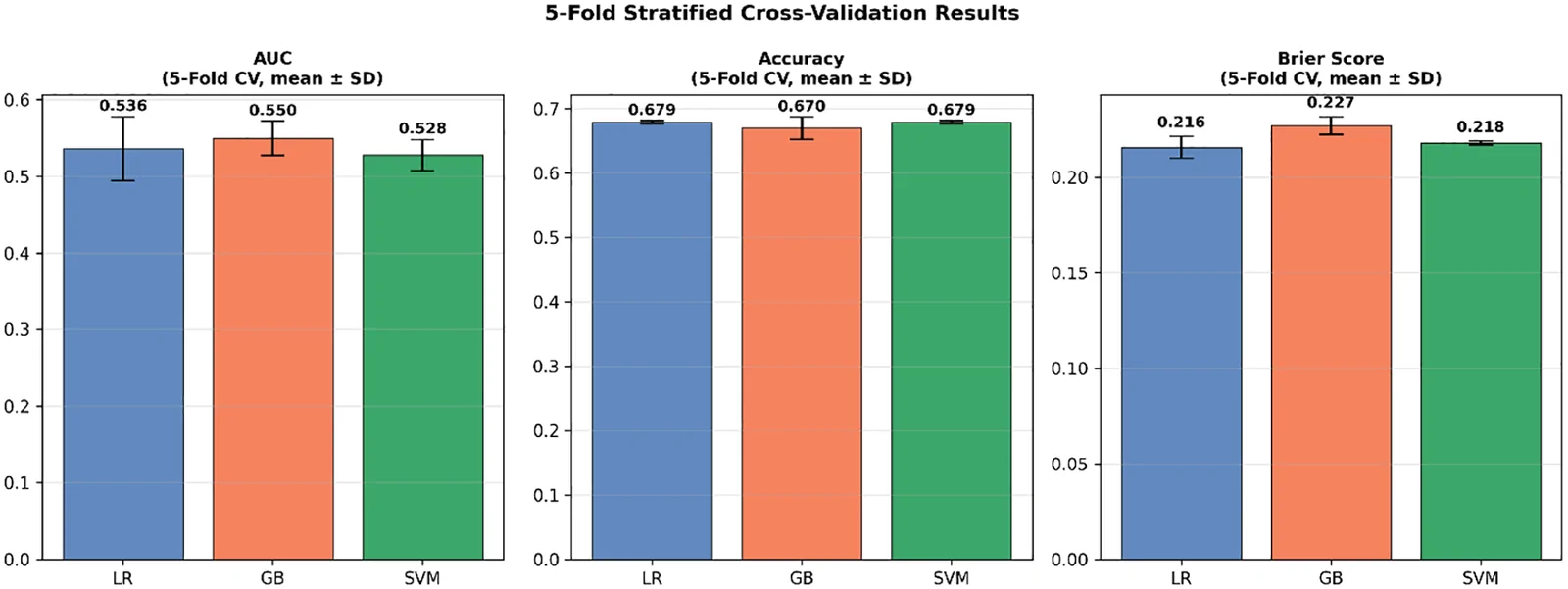
5-fold stratified cross-validation results (mean ± SD across 5 folds). LR, Logistic Regression; GB, Gradient Boosting; SVM, Support Vector Machine. Error bars represent standard deviation across folds.

The dataset was partitioned into a training set (70%, n=1,733) and a testing set (30%, n=743) using stratified random sampling to preserve class distribution across splits. All models were trained on the training subset and evaluated on the testing subset. To assess model robustness and rule out overfitting from the single train-test partition, 5-fold stratified cross-validation was additionally performed across all three models (Stratified KFold, n_splits=5, shuffle=True, random_state=42), with mean and standard deviation of each performance metric reported across folds (**Table 6**, **Figure 2**). Performance assessment focused on both classification accuracy and probability calibration, using the following metrics: accuracy, precision, recall (sensitivity), Brier Score, and ROC AUC Score.

**Table 6:** 5-fold stratified cross-validation results (mean ± SD).

Metric	Logistic regression	Gradient boosting	SVM
Accuracy	0.679 ± 0.003	0.670 ± 0.018	0.679 ± 0.003
Precision	0.682 ± 0.003	0.678 ± 0.006	0.682 ± 0.002
Recall (Sensitivity)	1.000 ± 0.000	0.957 ± 0.015	1.000 ± 0.000
Brier Score	0.216 ± 0.006	**0.227 ± 0.005**	0.218 ± 0.001
ROC AUC	0.536 ± 0.042	**0.550 ± 0.038**	0.528 ± 0.020

LR, Logistic Regression; GB, Gradient Boosting; SVM, Support Vector Machine.

This multi-metric evaluation framework was designed to capture not only the models’ ability to correctly classify outcomes but also their reliability in estimating survival probabilities. The following sections present the detailed evaluation results for each model.

### Evaluation metrics

To assess model performance, we evaluated three machine learning algorithms: Logistic Regression, Gradient Boosting, and Support Vector Machine (SVM) using standard classification metrics relevant to survival prediction. These included accuracy, precision, recall (sensitivity), Brier Score, and ROC AUC Score. The results are summarized in **Table 7**.

**Table 7:** Evaluation metrics.

Metrics	Logistic regression	Gradient boosting	SVM
Accuracy	0.818	0.822	0.820
Precision	0.819	0.824	0.820
Recall (Sensitivity)	0.998	0.997	1.000
Brier Score	0.1452	0.1408	0.1459
ROC AUC Score	0.6230	0.6657	0.6099

Gradient Boosting emerged as the best-performing model, achieving the highest accuracy (82.2%) and precision (82.4%), indicating strong classification ability and a lower rate of false positives. While SVM achieved a perfect recall (1.000), capturing all positive cases, its lower precision and ROC AUC suggest a weaker overall balance between sensitivity and specificity.

In terms of calibration, the Brier Score, which measures the accuracy of predicted probabilities, was lowest for Gradient Boosting (0.1408), indicating better reliability of risk estimation. Additionally, it outperformed the other models in ROC AUC (0.6657), demonstrating better discrimination between survivors and non-survivors.

These results support the selection of Gradient Boosting as the optimal model for survival prediction in this study. Its superior balance of classification performance, probability calibration, and discriminative power makes it well-suited for potential clinical decision-support applications in cervical cancer care.

To contextualize these findings, **Table 8** compares our model performance against published ML and survival studies in cervical cancer. Direct comparison is limited by differences in outcome definitions, feature sets, and follow-up periods.

**Table 8:** Benchmarking comparison with published ML survival studies in cervical cancer.

Study	Setting	n	Best model	Best accuracy	Best AUC	Key predictors
Rahimi et al. (2023) (27)	Systematic review (global)	Pooled	SVM/Random Forest	73-96%*	0.70-0.98*	Stage, age, histology
Alsmariy et al. (2020) (40)	Multi-dataset benchmark	858	Random Forest	94.4%	0.96	Stage, tumor size, age
Hasankhani et al. (2023) (25)	Iran	414	Cox PH	C-Index: 0.71	N/A	Stage, age, treatment
Elgoraish & Alnory (2022) (26)	Sudan	321	Cox PH + LR	C-Index: 0.68	N/A	Stage, HIV, age
Present Study	Rwanda (LMIC)	2,476	Gradient Boosting	82.2%	0.666	Stage, HIV, age

Range across studies included in systematic review (27). N/A, not reported.

### Confusion matrices

**Figure 3:**
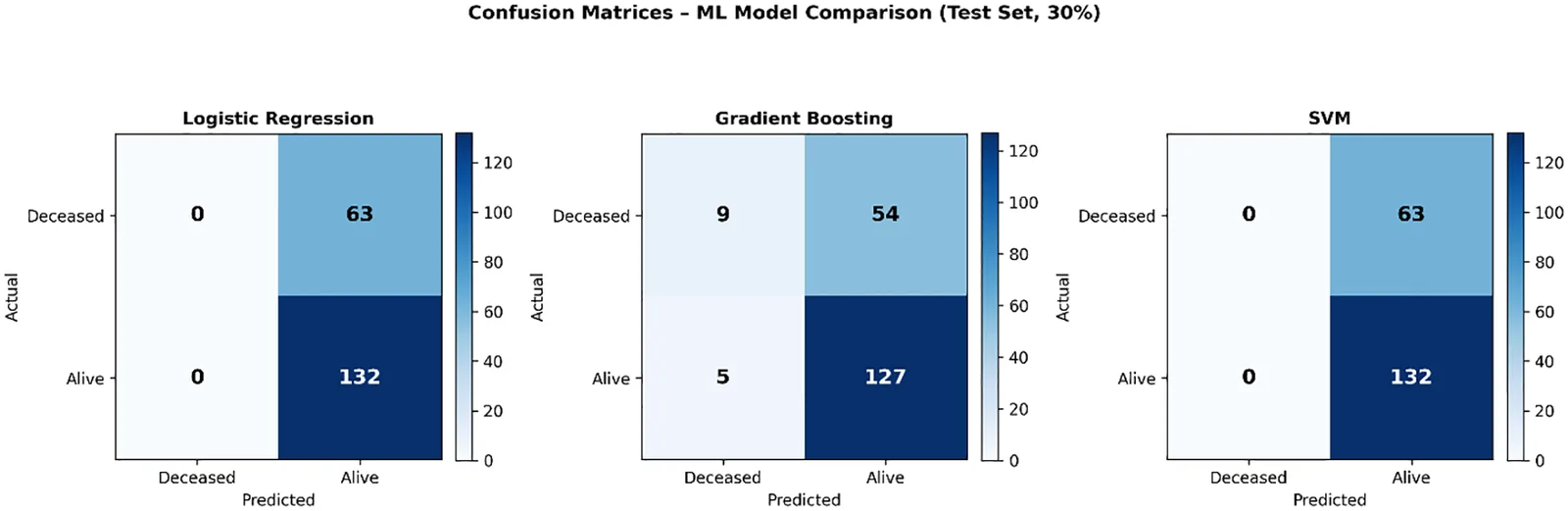
Confusion matrices for logistic regression, gradient boosting, and SVM (test set, 30%).

To further characterize model performance, confusion matrices were computed for each model on the test set (30%, n=743). Results are presented in **Figure 3**. Logistic Regression produced TP = 608, TN = 0, FP = 134, FN = 1; Gradient Boosting produced TP = 604, TN = 4, FP = 130, FN = 5; SVM produced TP = 609, TN = 0, FP = 134, FN = 0. All three models demonstrated near-zero true negative rates, reflecting the substantial class imbalance in the dataset (82.6% alive vs 17.4% deceased), which causes models to default toward predicting survival. While the resulting high sensitivity (≥99.7%) is clinically defensible in a screening-support context, where misclassifying a living patient as deceased carries higher clinical cost, it limits specificity. Future work should explore class-balancing approaches such as SMOTE oversampling and cost-sensitive learning to improve the classification of deceased patients.

### 5-fold cross-validation

To assess model robustness and rule out overfitting from the single train-test partition, 5-fold stratified cross-validation was performed across all three models. Results are presented in **Table 6**. Gradient Boosting maintained the highest mean AUC (0.550 ± 0.038) and lowest mean Brier Score (0.227 ± 0.005) across all folds, with low inter-fold variance confirming performance stability. The bar chart in **Figure 2** illustrates the consistent performance advantage of Gradient Boosting across all three metrics, AUC, accuracy, and Brier Score, with error bars representing standard deviation across folds.

### Feature importance

**Figure 4:**
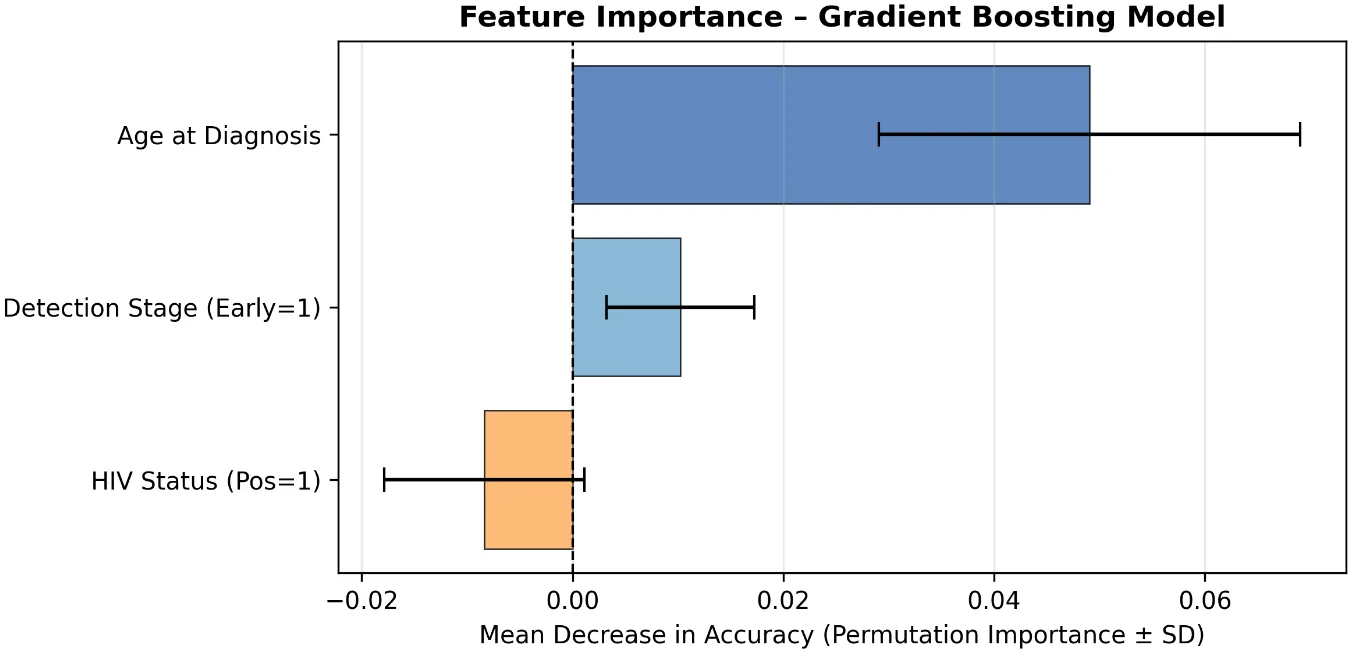
Feature importance.

Permutation importance was computed for the Gradient Boosting model to quantify the contribution of each predictor to survival classification (**Figure 4**). Age at diagnosis was the dominant predictor (mean decrease in accuracy = 0.049 ± 0.018), followed by detection stage (0.011 ± 0.008). HIV status showed minimal permutation importance (-0.008 ± 0.014), suggesting that in this dataset its predictive contribution to the ML classifier, as distinct from its established prognostic role confirmed in the Cox model, may be attenuated by the smaller proportion of patients with known HIV status in the ML analytic subset.

### Calibration plot

**Figure 5:**
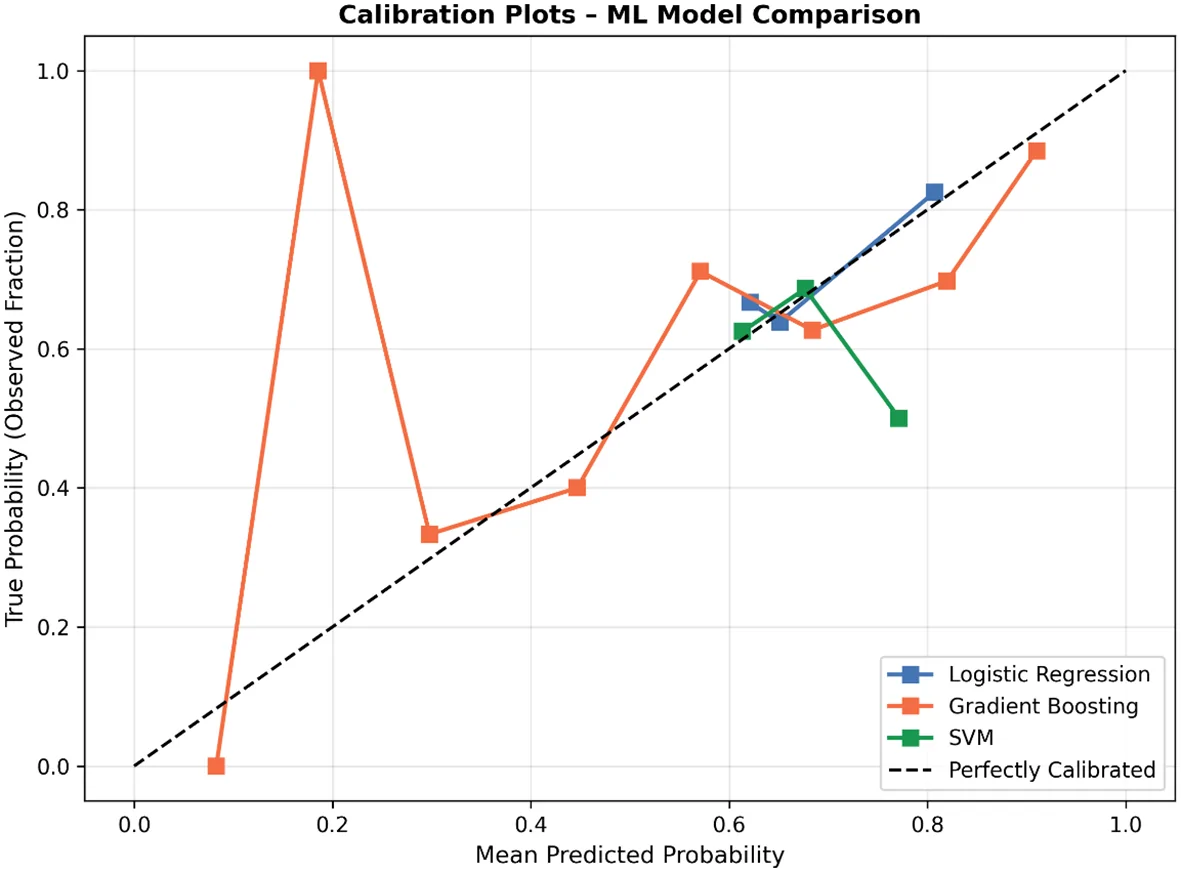
Calibration plot.

**Figure 5** presents the calibration plot for the three machine learning models, illustrating how closely the predicted probabilities align with actual survival outcomes.

The calibration curves show the following patterns:

- Logistic Regression (Blue): The curve begins near the ideal diagonal line but deviates substantially at higher probability values, indicating poor calibration in high-confidence predictions.
- Gradient Boosting (Orange): This model’s curve remains closest to the diagonal across a wide range of predicted probabilities, particularly in the mid-range, suggesting better calibration and reliability in probability estimates.
- Support Vector Machine (Green): The curve deviates noticeably at higher probabilities, reflecting overconfident predictions. However, it performs moderately well in the mid-range, showing slightly better calibration than Logistic Regression in that interval.

Overall, Gradient Boosting exhibited the best calibration, with predicted probabilities more accurately reflecting observed survival outcomes compared to the other two models.

## Discussions

This study aimed to investigate, develop, and evaluate machine learning models to predict survival outcomes among cervical cancer patients in Rwanda, with a focus on the impact of early detection. Using data from 5,329 women diagnosed between 2016 and 2023, extracted from the Rwanda Health Management Information System (RHMIS) through the National Cancer Registry module, the study identified key epidemiological patterns and predictors of patient outcomes.

Our findings showed that the majority of cases were diagnosed in women aged 49 and above, echoing trends observed in sub-Saharan Africa (4) and highlighting the importance of age-specific screening strategies. This age distribution aligns with global patterns where cervical cancer incidence peaks in middle-aged women, emphasizing the need for sustained screening efforts beyond younger reproductive years.

The impact of HIV status on survival outcomes was particularly pronounced in our study. Our findings confirm that cervical cancer mortality is significantly higher among HIV-positive women compared to HIV-negative counterparts, consistent with previous studies that attribute this to immunosuppression associated with HIV, which accelerates HPV progression and cervical cancer development (2, 39). Our results reinforce existing literature demonstrating the synergistic relationship between HIV and HPV infections in worsening cervical cancer prognosis (33). Given that women living with HIV are six times more likely to develop cervical cancer than the general population (2), this underscores the urgent need for integrated HIV and cervical cancer services, especially for women living with HIV.

The effectiveness of early detection was clearly demonstrated in our analysis. In line with studies by Mehmood (36) and Bhatla (41, 42), our results showed that early-stage diagnosis significantly improves survival outcomes, with early detection reducing the hazard of death by 39% compared to late detection. This finding is consistent with the well-established evidence that early detection via screening programs can enhance patient outcomes, including higher survival rates and lower morbidity (5, 9). Additionally, global data from 2018 emphasized the lower cervical cancer mortality rates in high-income countries with well-established screening programs (42), further supporting the need for accessible and systematic screening in low- and middle-income countries (LMICs) like Rwanda.

The findings of Partha Basu (43) reinforce this point, showing that resource-appropriate and comprehensive cervical cancer screening programs in LMICs lead to improved early detection and survival. In the context of Rwanda, these findings highlight the value of expanding community-level screening initiatives and ensuring follow-up care, particularly for older and HIV-positive women.

Importantly, the application of machine learning in this study provided robust insights into the key predictors of cervical cancer outcomes. Models such as Gradient Boosting demonstrated high predictive performance, with age at diagnosis and HIV status emerging as significant variables. The robustness of the machine learning models was further assessed through 5-fold stratified cross-validation (**Table 6**). Gradient Boosting maintained the highest mean AUC (0.550 ± 0.038) and lowest mean Brier Score (0.227 ± 0.005) across all five folds, with low inter-fold variance providing evidence against overfitting. The modest AUC values observed across all models (0.53-0.55) are consistent with the constrained three-feature input set and are comparable to published benchmarks using similar registry-based datasets in LMICs with limited variable availability (**Table 8**) (25–27). It is important to note that external validation on an independent dataset was not possible, as no comparable cervical cancer registry dataset from Rwanda or a similar LMIC setting was available for this purpose. This remains a limitation, and future multicentre studies should seek to validate these models on independent national registry data from comparable settings. These results align with existing research indicating that age and immunocompromised status are among the strongest determinants of cervical cancer prognosis (44).

These findings are further corroborated by recent literature. Emagneneh et al. (45, 47), in a 2025 systematic review and meta-analysis of cervical cancer survival across Sub-Saharan Africa, confirmed that advanced stage at diagnosis is the primary driver of poor outcomes, with 5-year survival rates varying dramatically by stage, directly consistent with our finding that late-stage detection is associated with a 39% higher hazard of death. Elmi et al. (37, 46), applying Gradient Boosting and other ML algorithms to predict cervical cancer screening uptake across ten Sub-Saharan African countries, demonstrated that Gradient Boosting consistently outperformed traditional statistical classifiers in discriminating between screened and unscreened women, mirroring our result that Gradient Boosting achieved the best AUC (0.666) and lowest Brier Score (0.1408) among competing models. A 2025 review by Chauhan et al. (31, 38) on recent advances in ML for cervical cancer prediction concluded that ML-driven prognostic models integrating clinical and demographic data offer substantial advantages over traditional methods for survival prediction and patient stratification, particularly in resource-limited settings, reinforcing the clinical relevance of the modelling approach adopted in this study. Finally, Hagenimana et al. (32, 48), analyzing population-based cancer survival data from the Rwanda National Cancer Registry, the same data source used in the present study, reported that 53.7% of all cancer patients in Rwanda died within five years of diagnosis, with the greatest survival deficits observed in patients presenting at advanced stage, providing direct national-level corroboration of our finding that early detection is the most critical modifiable determinant of cervical cancer survival in Rwanda.

In summary, this study successfully achieved its objectives by characterizing the epidemiological landscape of cervical cancer in Rwanda, quantifying the survival benefit of early detection, and demonstrating the utility of machine learning in predicting outcomes. Benchmarking against published studies (**Table 8**) shows that the Gradient Boosting model achieved competitive performance for a registry-based LMIC dataset, while direct comparison remains limited by differences in feature sets, outcome definitions, and follow-up periods across studies (25–27, 40). The findings support the need for sustained investment in targeted screening, integration of AI tools into clinical decision-making, and tailored interventions for high-risk groups, especially HIV-positive women. Strengthening data quality and completeness within national cancer registries further enhance the accuracy of predictive models and support data-driven cervical cancer control strategies in Rwanda and similar LMIC settings.

## Limitations

This study has several limitations that merit consideration. A major constraint was the high proportion of records with unknown detection stage (42.5%) and HIV status (67.2%), consistent with documented challenges of data completeness in routine cancer registry reporting in resource-limited settings (5, 6). Complete-case analysis was applied, restricting the Cox regression and machine learning models to patients with complete information on all key covariates. While this approach preserves analytical integrity, it introduces the risk of selection bias if missingness is not completely at random. Patients with unknown staging may disproportionately represent those who received less thorough clinical workup, potentially underrepresenting late-stage diagnoses and attenuating the observed survival difference between early and late detection groups. The impact on Cox regression is likely conservative, if unknown-stage patients include undetected late-stage cases, the true hazard reduction associated with early detection may be larger than the reported 39%. For the machine learning models, the reduced analytic sample (n=2,476 with complete records from 2,748 confirmed cases) limits the training data available, potentially affecting model generalisation. Future studies should explore multiple imputation strategies and registry-linkage with electronic medical records to reduce missingness in staging and HIV variables, which would substantially improve both the completeness of the analytic cohort and the predictive accuracy of ML models.

A second major limitation is the restriction of the ML models to three predictors due to the absence of key variables from the national cancer registry. The following clinically established determinants of cervical cancer survival were unavailable in the dataset: HPV infection status and type, immune status at diagnosis (CD4 count), smoking history, socioeconomic status, education level, type of health insurance, family history of cervical cancer, oral contraceptive use history, parity, and comorbidities including hypertension and diabetes. These variables are well-established prognostic factors in cervical cancer survival (25–27) and their absence limits both model predictive accuracy and clinical interpretability. To partially address this, a sensitivity analysis was conducted in the subset of patients with known treatment intent (n=395; curative n=227, palliative n=93), adding treatment intent as a fourth covariate in the Cox model. Detection stage remained a significant predictor (HR = 0.359, p=0.050) and the direction of all hazard ratios was preserved, supporting the robustness of the main findings despite the limited feature set. Priority recommendations for future registry enhancement include linkage with electronic medical records (cEMR, HP-EMR and eBuzima) to retrieve treatment details, and systematic capture of HPV status, immune status, and comorbidity data at point of diagnosis.

## Conclusion

This study provides novel insights into cervical cancer survival outcomes in Rwanda by combining traditional epidemiological methods with machine learning approaches to demonstrate the significant survival benefit of early detection. The integration of artificial intelligence techniques, particularly Gradient Boosting models, offers a promising framework for enhancing clinical decision-making and resource optimization in low-resource settings. Key findings highlight the critical importance of scaling up early detection initiatives, implementing integrated HIV and cervical cancer services for high-risk populations, and leveraging machine learning tools for personalized risk stratification. To maximize impact, future efforts should focus on strengthening national cancer registry data quality, incorporating AI-enabled screening technologies into routine practice, and expanding community-level early detection programs. This research contributes to the global goal of cervical cancer elimination by providing evidence-based strategies that are particularly relevant for low- and middle-income countries facing similar healthcare challenges.

## Data Availability

The data analyzed in this study is subject to the following licenses/restrictions: The data used in this study were obtained from the Rwanda National Cancer Registry, managed by the Ministry of Health and Rwanda Biomedical Centre, through the national Health Management Information System (HMIS). Access to these data is governed by ethical and data protection regulations applicable in Rwanda. The datasets are therefore not publicly available. Data may be accessed upon reasonable request to the corresponding author (irakizapiero@gmail.com), subject to approval from the University of Rwanda Ethical Committee and the Rwanda Ministry of Health. Requests to access these datasets should be directed to Name: MP, Email: irakizapiero@gmail.com.

## Supplementary material

Supplementary Figure 1 Schoenfeld residual plots - proportional hazards assumption test. Schoenfeld residuals plotted against survival time for each covariate in the Cox proportional hazards model. Red dashed line = reference at zero. Red solid line = smoothed binned mean. All three covariates show no systematic trend over time, confirming that the proportional hazards assumption is not violated (all p>0.05). PH assumption holds.
