# Supplementary material for: Leveraging machine learning algorithms to assess the impact of cervical cancer early detection on patients’ survival
Source: Front Oncol. 2026 Sep 1;16:1880629. doi: 10.3389/fonc.2026.1880629 (PMC13574586; doi:10.3389/fonc.2026.1880629)
Supplement: Supplementary file 1 [file Table1.docx]

**Supplementary Table S1**: Supplementary Table S1: Comparison of Baseline Characteristics Between Included and Excluded Confirmed Cervical Cancer Patients (2016-2023)

| **Characteristic** | **Included (n=2,476)** | **Excluded (n=272)** | **p-value** |
| --- | --- | --- | --- |
| Age at diagnosis, mean±SD (years) | 54.2 ± 12.2 | 55.1 ± 12.8 | 0.241+ |
| **Survival status** | | | |
| Alive | 2,045 (82.6%) | 224 (82.4%) | 0.918# |
| Deceased | 431 (17.4%) | 48 (17.6%) |  |
| **Detection stage** | | | |
| Early | 183 (7.4%) | 14 (5.1%) | — |
| Late | 1,241 (50.1%) | 98 (36.0%) |  |
| Unknown | 1,052 (42.5%) | 160 (58.8%) |  |
| **HIV status** | | | |
| Negative | 622 (25.1%) | 48 (17.6%) | — |
| Positive | 191 (7.7%) | 12 (4.4%) |  |
| Unknown | 1,663 (67.2%) | 212 (77.9%) |  |
| **Treatment intent** | | | |
| Curative | 651 (26.3%) | 52 (19.1%) | 0.089# |
| Palliative | 337 (13.6%) | 23 (8.5%) |  |
| Unknown | 1,488 (60.1%) | 197 (72.4%) |  |

*+Independent samples t-test. # Chi-square test (Alive vs Deceased only, excluding Unknown category). Not tested: unknown category is the structural reason for exclusion. Values are n (%) unless stated otherwise.*
